# Progress of the Use of Chloroform

**Published:** 1848-05

**Authors:** 


### PROGRESS OF THE USE OF CHLOROFORM.

We continue to give under this head all the accounts published of the injurious effects of the new anœsthetic; the cases in which it has been used beneficially if published would fill our Journal.

"It is stated in the New York Express, that Mrs. Rehlsant was put under the influence of chloroform, for the purpose of having a tooth extracted. After inhaling the article, she lay lifeless for several hours, and was finally conveyed home in a state of insensibility; ultimately she recovered her mental faculties, but has since been laboring under prostration, paralysis of the tongue, and loss of voice.

"A young man in New Bedford, who inhaled chloroform for amusement, is said in the papers to have been thrown into convulsions which lasted for sixteen hours without intermission.

"In Baltimore, a medical student came near losing his life from the same cause; after inhaling chloroform for a time, he became insensible, in which state he remained one hour and a half.

"An apothecary's apprentice in Philadelphia was thown into convulsions by the inhalation of chloroform.

"A lady of Cincinnati, in the enjoyment of good health, who inhaled chloroform, preparatory to having a tooth extracted, suddenly sunk under its effects and did not revive.

"A man in New York, suddenly expired after inhaling chloroform to produce insensibility to the pain of an operation for fistula in ano."—*Medical News.*

